# Supplementary figures and images for: Synergistic Effects of the Jackfruit Seed Sourced Resistant Starch and Bifidobacterium pseudolongum subsp. globosum on Suppression of Hyperlipidemia in Mice
Source: Foods. 2021 Jun 21;10(6):1431. doi: 10.3390/foods10061431 (PMC8235523; doi:10.3390/foods10061431)

A

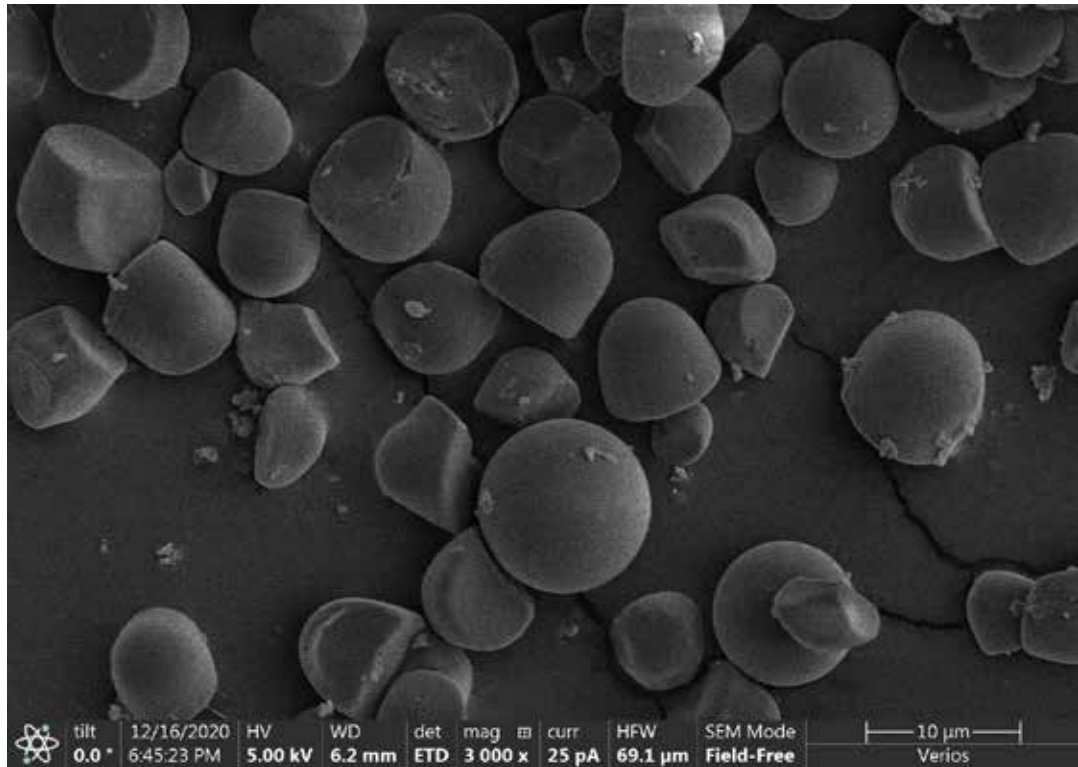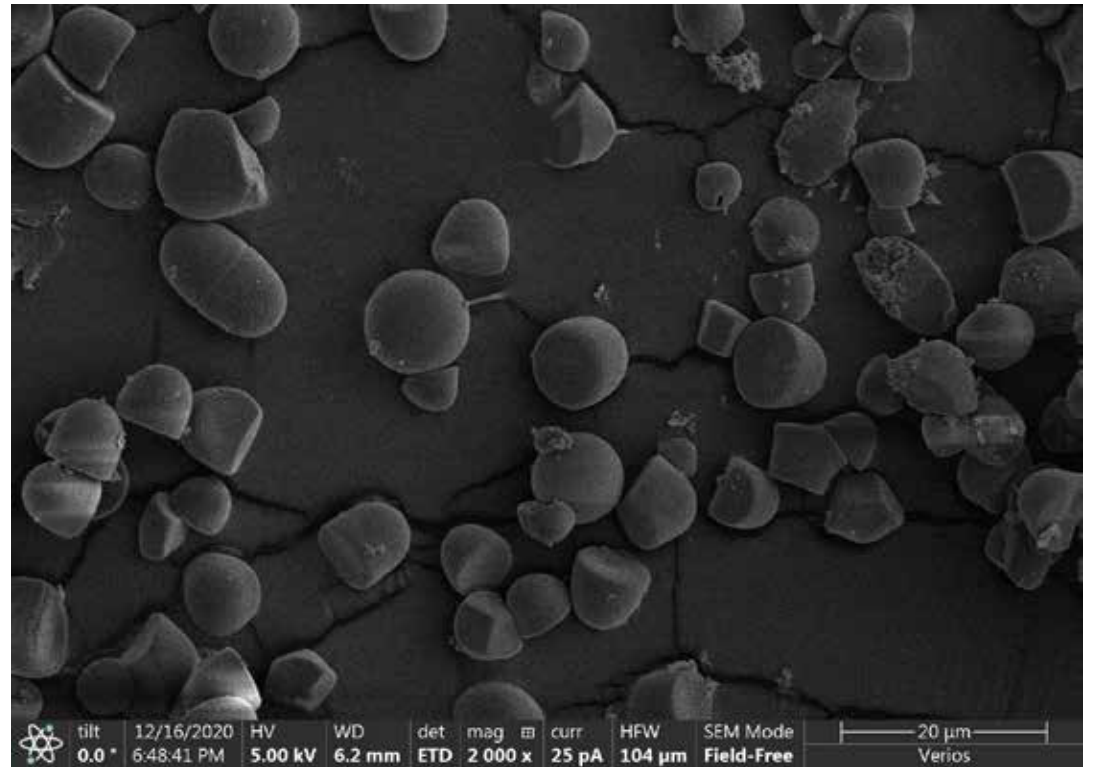

B

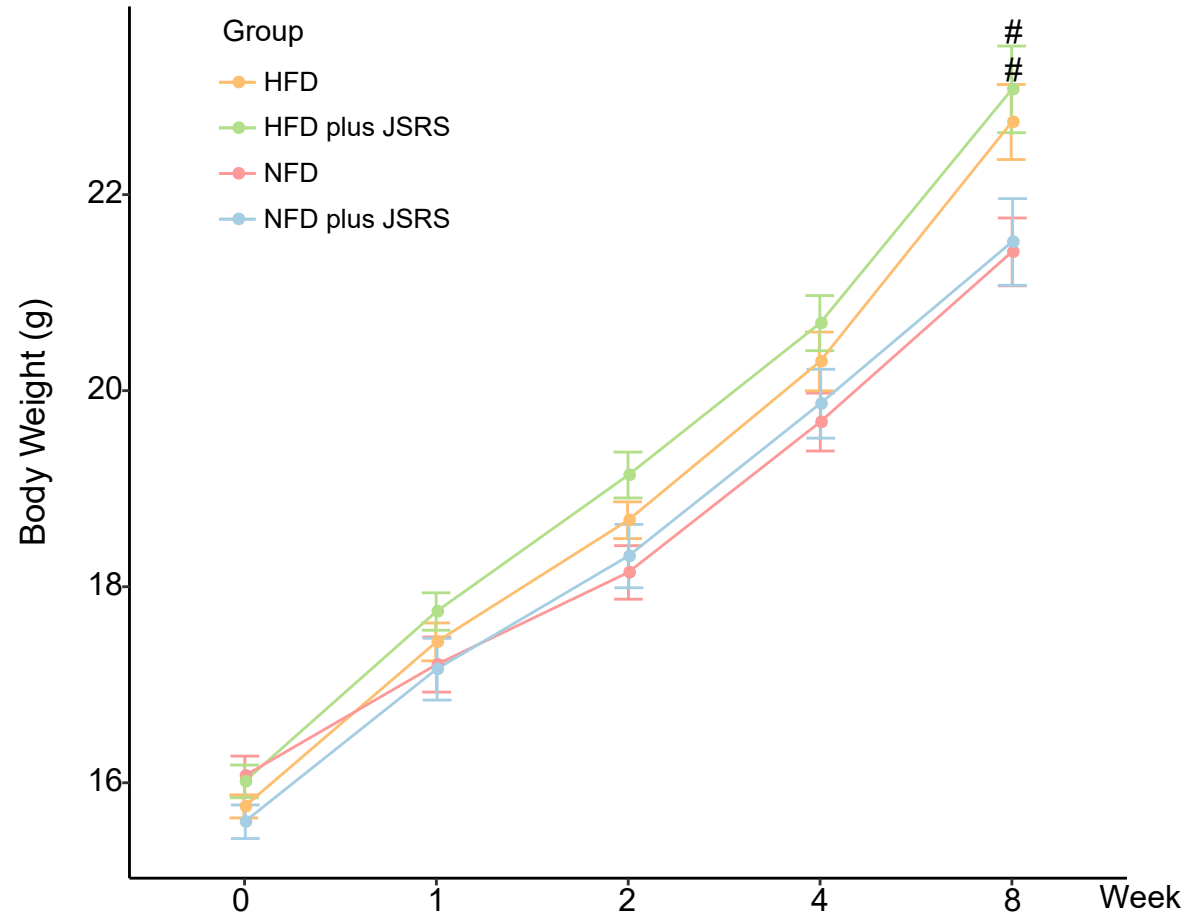

C

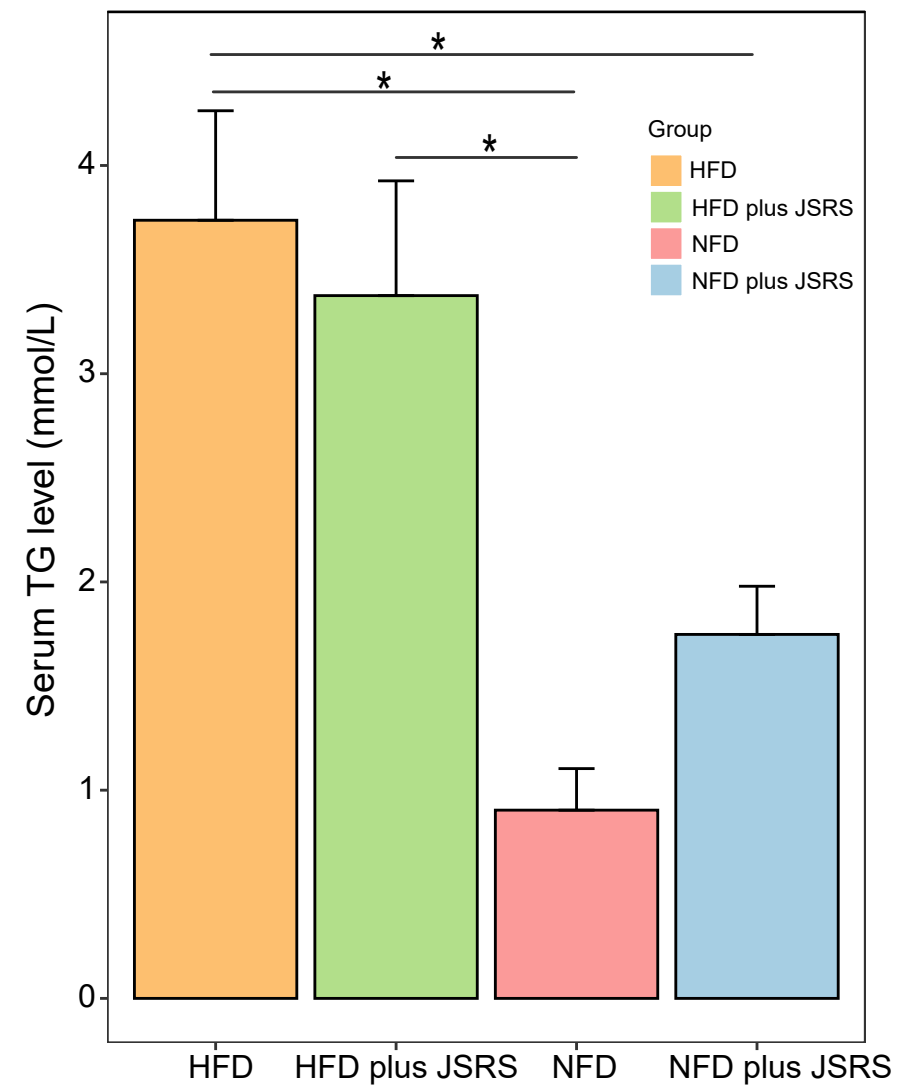

Supplement: Supplementary file 1 [file foods-10-01431-s001.zip › Supplemental Figure S1.pdf]

**A**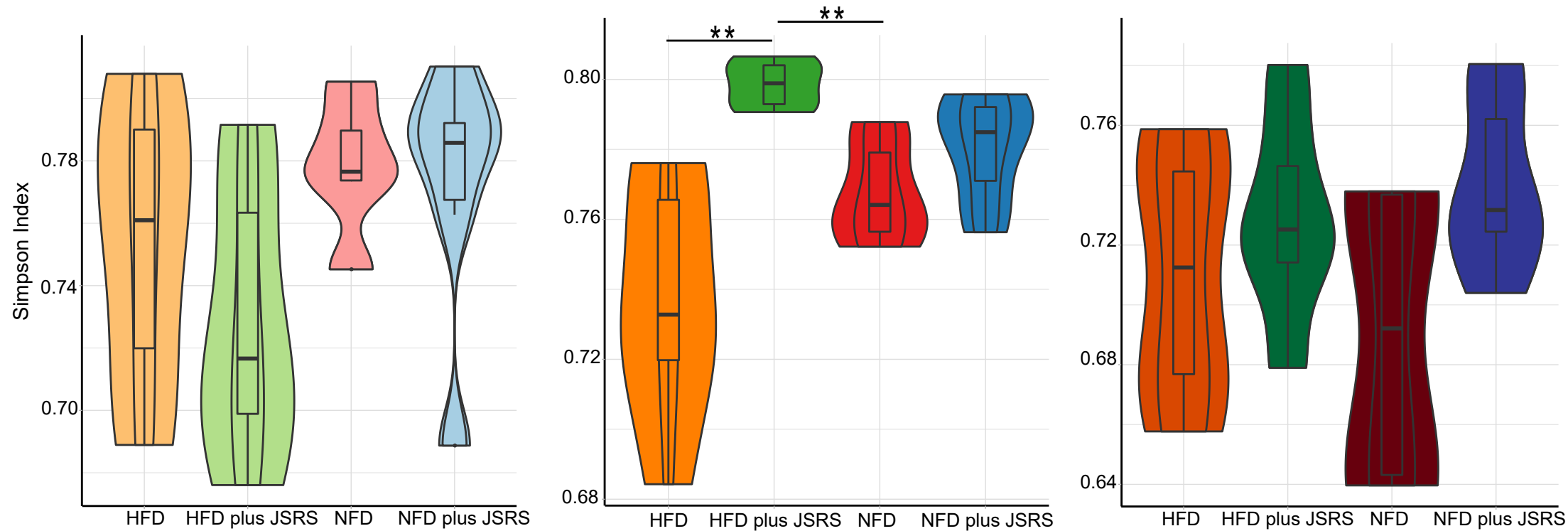**B****Week 2****Week 4****Week 8**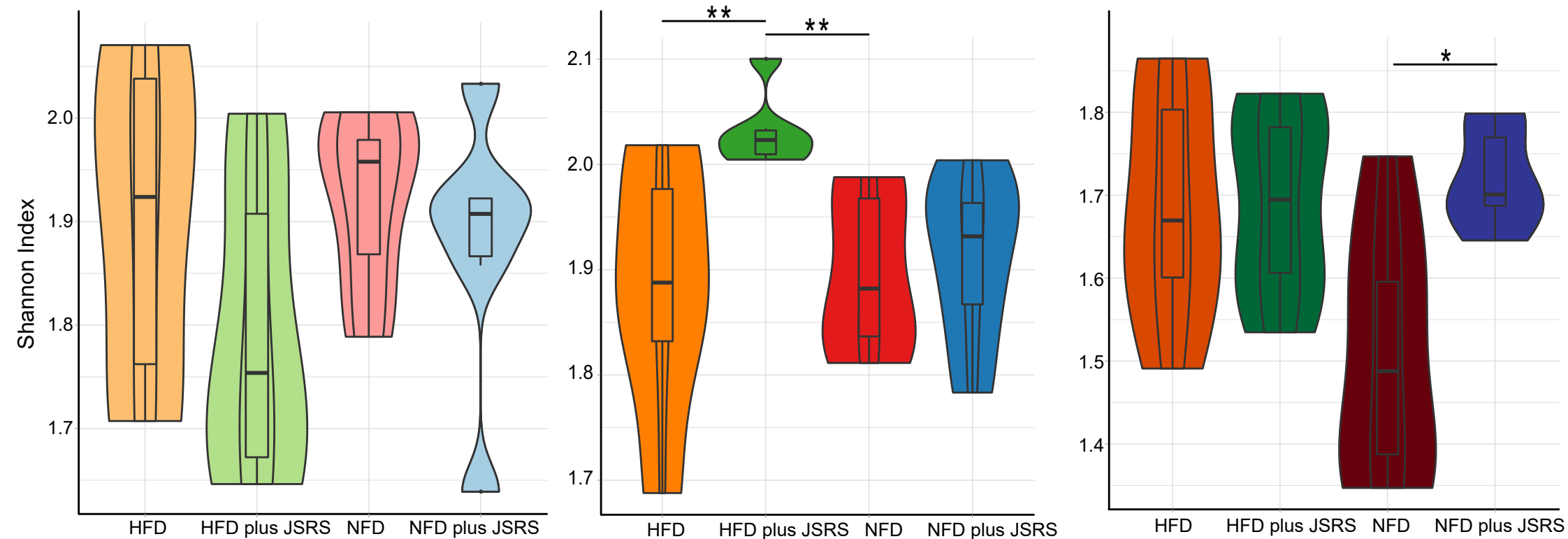

Supplement: Supplementary file 1 [file foods-10-01431-s001.zip › Supplemental Figure S2.pdf]

A

JSRS medium (After the iodine solution added)

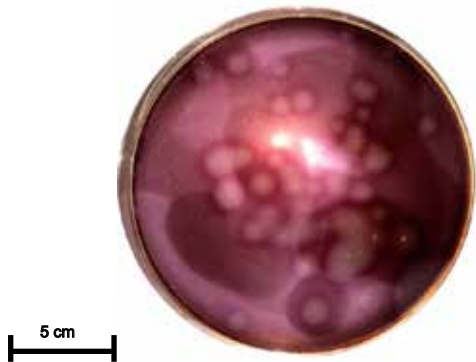

B

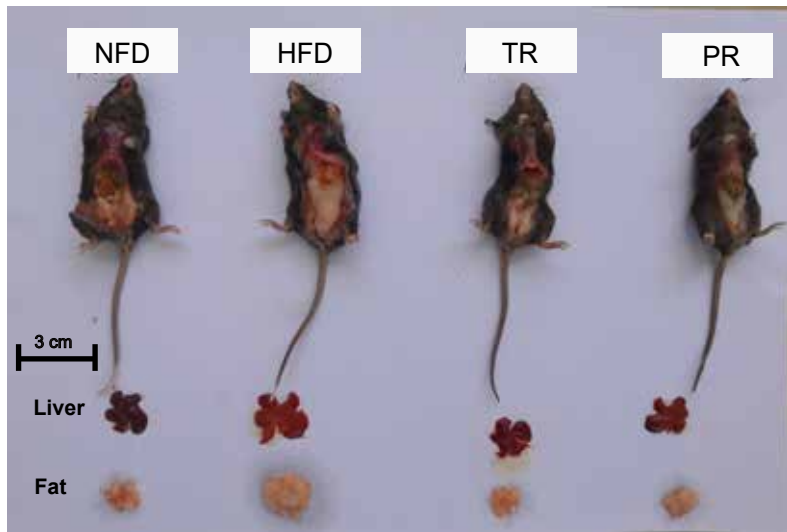

Supplement: Supplementary file 1 [file foods-10-01431-s001.zip › Supplemental Figure S3.pdf]
